# A Nomogram Model that Predicts the Risk of Diabetic Nephropathy in Type 2 Diabetes Mellitus Patients: A Retrospective Study

**DOI:** 10.1155/2021/6672444

**Published:** 2021-04-08

**Authors:** Chunfeng Xi, Caimei Wang, Guihong Rong, Jinhuan Deng

**Affiliations:** Department of Laboratory Medicine, Affiliated Hospital of Guilin Medical University, Guilin, Guangxi, China

## Abstract

**Objective:**

To construct a novel nomogram model that predicts the risk of diabetic nephropathy (DN) incidence in Chinese patients with type 2 diabetes mellitus (T2DM).

**Methods:**

Questionnaire surveys, physical examinations, routine blood tests, and biochemical index evaluations were conducted on 1095 patients with T2DM from Guilin. A least absolute contraction selection operator (LASSO) regression and multivariable logistic regression analysis were used to screen out DN risk factors. A logistic regression analysis incorporating the screened risk factors was used to establish a predictive nomogram model. The performance of the nomogram model was evaluated using the C-index, an area under the receiver operating characteristic curve (AUC), calibration plots, and a decision curve analysis. Bootstrapping was applied for internal validation.

**Results:**

Independent predictors for DN incidence risk included gender, age, hypertension, medicine use, duration of diabetes, body mass index, blood urea nitrogen level, serum creatinine level, neutrophil to lymphocyte ratio, and red blood cell distribution width. The nomogram model exhibited moderate prediction ability with a C-index of 0.819 (95% confidence interval (CI): 0.783–0.853) and an AUC of 0.813 (95%CI: 0.778–0.848). The C-index from internal validation reached 0.796 (95%CI: 0.763–0.829). The decision curve analysis displayed that the DN risk nomogram was clinically applicable when the risk threshold was between 1 and 83%.

**Conclusion:**

Our novel and simple nomogram containing 10 factors may be useful in predicting DN incidence risk in T2DM patients.

## 1. Introduction

Type 2 diabetes mellitus (T2DM) is a progressive metabolic disorder; its main clinical manifestation is hyperglycemia. Over the past decade, the global occurrence of T2DM has dramatically increased. According to a report by the International Diabetes Federation (IDF), the prevalence of diabetes was 382 million in 2013, 415 million in 2015, and 425 million in 2017. In 2019, the IDF Diabetes Atlas reported that approximately 463 million adults (20–79 years old) worldwide live with T2DM. In China, only 0.67% of the population had diabetes in 1980 [1]. However, from 2010 to 2017, the prevalence of diabetes had rapidly increased to 9–12% [2–4].

Many individuals with T2DM readily develop complications, particularly diabetes nephropathy (DN) [5]. Not only is DN one of the most common and severe chronic microvascular complications of T2DM, it is also the main cause of end-stage renal failure and death in diabetic individuals. In a cross-sectional study from 2014 to 2015 involving 5,078 community outpatients with T2DM, the prevalence of DN was 38.4% [6]. In Africa, the incidence of end-stage renal disease in diabetic patients was 34.7% at a 5-year follow-up, while the mortality of DN was 18.4% at a 20-year follow-up [7]. In the United States, approximately 30–40% of diabetic patients suffer from DN [8]. The increasing prevalence of diabetes, with DN, will inevitably become a major health burden on health systems in both developed and developing countries.

The creation of a DN incidence risk nomogram can be beneficial for early intervention and, consequently, improve the quality of life of diabetic patients. Until now, several predictive nomogram models have been reported to evaluate diabetic complications, such as DN, diabetic retinopathy, and diabetic foot [9–14]. However, the above nomogram models do not include inflammation-related factors. Therefore, the aim of this study was to build a simple and practical nomogram model that incorporates inflammation-related factors in evaluating DN risk in T2DM patients.

## 2. Materials and Methods

### 2.1. Patients

This project was conducted retrospectively from January 2019 to January 2020 in Guilin. It was approved by the Affiliated Hospital of Guilin Medical University Ethics Committee, and each patient gave written informed consent.

Baseline data from T2DM patients was obtained from the electronic medical record system of the Affiliated Hospital of Guilin Medical University. T2DM was diagnosed based on criteria defined by the World Health Organization in 1999. The diagnostic criterion for DN was urinary albumin to creatinine ratio greater than 30 mg/g. To be included, subjects must have been diagnosed with T2DM, were over 18 years old and either had a local household registration or had lived in the community for at least 6 months. They were excluded if they had any severe chronic disease, including chronic pulmonary heart disease, chronic aplastic anemia, liver failure, cirrhosis, systemic lupus erythematosus, myocardial infarction or severe heart failure, uremia dialysis, or a malignant tumor.

Altogether, 1,095 subjects were enrolled in this study. They were divided into the DN (203) and nondiabetic nephropathy (NDN; 892) groups.

### 2.2. Investigation Methods

All participants underwent a questionnaire survey, a physical examination, a routine blood test, and a biochemical index evaluation. The questionnaire survey collected each patient's basic information, including gender, age, daily lifestyle habits (i.e., alcohol consumption and smoking), medication usage, and duration of diabetes. The physical examination measured the patient's height, weight, and blood pressure; body mass indices (BMI) were calculated. Each patient's fasting blood was collected in the morning. The patients' postprandial blood was collected two hours after breakfast. The routine blood test evaluated white blood cell (WBC), neutrophil (N), and lymphocyte (*L*) counts; the neutrophil to lymphocytes ratio (NLR), and the red blood cell distribution width (RDW). The biochemical index evaluation assayed levels of glycosylated hemoglobin A1c (HbA1c), triglycerides (TG), total cholesterol (TC), high-density lipoprotein cholesterol (HDL), fasting blood glucose (FBG), serum creatinine (SCr), blood urea nitrogen (BUN), uric acid (UA), high sensitivity C-reactive protein (Hs-CRP), and postprandial blood glucose (PBG).

### 2.3. Statistical Analysis

All data are described as frequency counts (percentages). *R* software (version 3.6.2; https://www.R-project.org) was used for the statistical analysis.

The least absolute contraction selection operator (LASSO) was used to screen optimal predictors among the current risk factors in patients with DN. This method is ideal in reducing high-dimensional data. Features with regression coefficients equal to zero were removed from the LASSO regression model, while those with nonzero regression coefficients were retained. From the remaining predictors, a multivariable logistic regression analysis was applied, and the predictors that were statistically significant were selected. Finally, the statistically significant predictors were used to construct a nomogram model to predict DN incidence risk in T2DM patients.

To evaluate the discrimination performance of the DN incidence risk nomogram, a C-index examination was conducted. Bootstrap samples (1000 bootstrap resamples) were randomly selected from the original samples to use as an internal validation for a corrected C-index. An area under the receiver operating characteristic curve (AUC) was applied to judge the ability of the DN risk nomogram in discriminating true positives from false positives. To evaluate its calibration capabilities, calibration curves were drawn. A decision curve analysis defined the clinical practicability of the DN risk nomogram by calculating its net benefits based on different threshold probabilities in patients with T2DM.

## 3. Results

### 3.1. Patients Characteristics

A total of 1,095 patients with T2DM, 472 females and 623 males, were included in this study. The patients were divided into DN and NDN groups based on DN diagnostic criterion. Patient baseline characteristics between the two groups, such as demographics, physical examination results, routine blood test parameters, and biochemical indices are shown in Table 1.

### 3.2. Feature Selection

**Figure 1:**
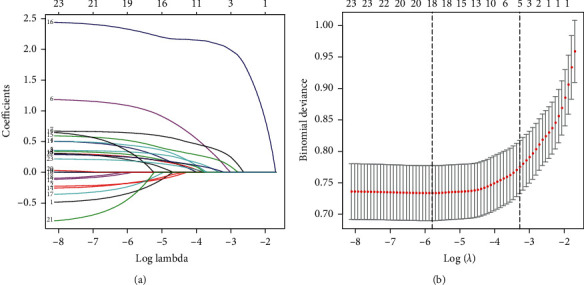
Clinical feature selection using the LASSO binary logistic regression model. (a) A coefficient profile plot was drawn against the log (lambda) sequence. (b) Eighteen features with nonzero coefficients were screened by optimal lambda. The partial likelihood deviance (binomial deviance) curve was drawn versus log (lambda). Dotted vertical lines were plotted according to 1 standard error criteria.

From the baseline characteristics, 18 potential predictors out of the 23 clinical features had nonzero coefficients in the LASSO regression model (Figures 1(a) and 1(b)). Then, 10 potential predictors were further selected using the multivariable logistic regression analysis. The above potential predictors included gender, age, hypertension, medication use (oral medicine and/or insulin injection), duration of diabetes, BMI, BUN, SCr, NLR, and RDW.

### 3.3. Construction of the Nomogram Prediction Model

**Figure 2:**
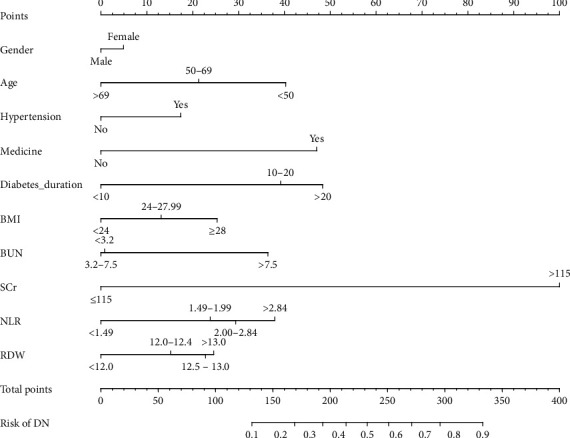
Constructed DN incidence risk nomogram. The DN risk nomogram was constructed with the features, including gender, age, hypertension, medication use, duration of diabetes, BMI, BUN, SCr, NLR, and RDW. DN, diabetic nephropathy; BMI, body mass indices; BUN, blood urea nitrogen; SCr, serum creatinine; NLR, neutrophil to lymphocytes ratio; RDW, red blood cell distribution width.

The results of the multivariable logistic regression analysis among gender, age, hypertension, medication use, duration of diabetes, BMI, BUN, SCr, NLR, and RDW are shown in Table 2. Statistically significant differences were observed among these 10 predictors. The 10 independent predictors (gender, age, hypertension, medication use, duration of diabetes, BMI, BUN, SCr, NLR, and RDW) were used to build the prediction model (Figure 2).

### 3.4. Performance of the DN Risk Nomogram in the Cohort

**Figure 3:**
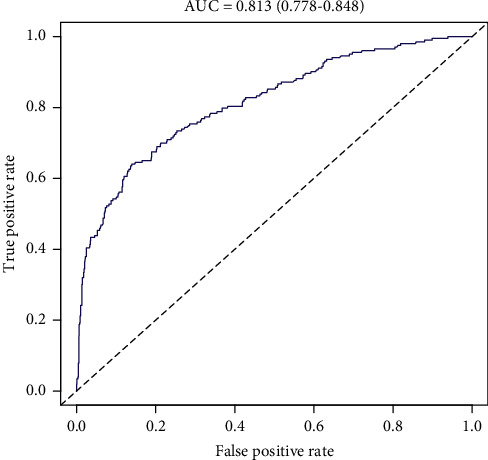
The AUC of the DN risk nomogram model. The *x*-axis represents the false positive rate of the risk prediction, while the *y*-axis represents the true-positive rate of the risk prediction. The blue line displays the performance of the nomogram.

**Figure 4:**
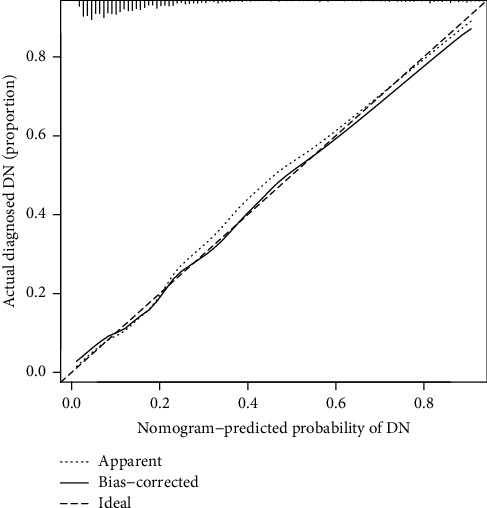
Calibration curves of the DN risk nomogram prediction. The diagonal dotted line meant a perfect prediction by an ideal model while the solid line represented the performance of the nomogram. A closer fit to the diagonal dotted line meant a better prediction.

The C-index of the nomogram in predicting DN risk was 0.819 (95% confidence interval: 0.783–0.853) and was confirmed to be 0.796 (95%CI: 0.763–0.829) by internal validation, indicating that the model's performance was moderate. The AUC was 0.813 (95%CI: 0.778–0.848, Figure 3), demonstrating good discrimination. For the 1,095 T2DM patients in the study, the calibration of the nomogram in forecasting DN risk displayed good agreement (Figure 4). Altogether, our nomogram prediction model showed medium predictive capability.

### 3.5. Clinical Use

**Figure 5:**
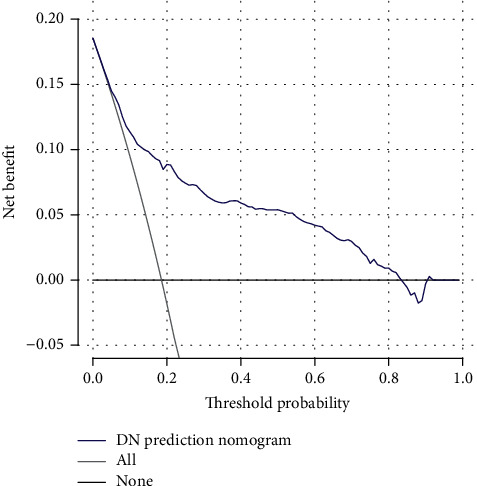
Decision curve analysis for the DN risk nomogram. The *y*-axis tested the net benefit. The thin solid line meant the assumption that all patients had DN, while the thick solid line represented the assumption that all patients had no DN. The dotted line represented the risk nomogram.

The decision curve of the DN risk nomogram is plotted in Figure 5. According to the decision curve analysis, the utility of the nomogram in predicting DN incidence risk is more beneficial than preexisting schemes when DN risk threshold probabilities are between 1 and 83%.

## 4. Discussion

Until now, very few comprehensive nomogram models that predict DN risk in T2DM patients are available. Nomograms constructed from only demographic data and biochemical test results often lack good predictive abilities. Biochemical and routine blood tests are common assays in diagnosis and treatment. The logistic regression analysis in our study found that gender, age, hypertension, medication use, duration of diabetes, BMI, BUN, SCr, NLR, and RDW were independently associated with DN incidence risk. This study is the first to incorporate these indicators, especially the inflammation-related NLR and RDW, in building a nomogram to predict DN incidence risk among T2DM patients. The high C-index in both training set and internal verification confirmed that the nomogram has high accuracy as well as good predictive ability.

The occurrence and development of DN in T2DM patients involved multiple factors. A large pediatric population survey of T2DM patients found that the prevalence of DN was lower in male youths compared to females [15]. Similarly, a systematic review and meta-analysis reported that the risk of end-stage renal disease in male diabetics was lower than in females [16]. Moreover, several studies have identified the female sex as an independent predictor for adverse DN [17, 18]. The results of our study are in accordance with these reports mentioned above. Additionally, numerous studies have found that younger age, medication use (oral medicine and/or insulin injection), and longer duration of diabetics strongly associate with DN [19–21]. A retrospective study of 220 Chinese patients with T2DM showed that age, duration of diabetes, and systolic blood pressure were independently correlated with DN incidence risk [22]. Another retrospective survey involving 11,771 participants with T2DM revealed that younger age, higher BMI, and more severe hypertension were independent risk factors that can increase DN incidence [23]. Consistent with these reports, our results from the multivariable logistic regression analysis revealed that younger age, longer duration of diabetes, medication use, higher BMI, and higher hypertension were significantly associated with increased DN risk. An explanation for our results is that the younger age of diabetes likely increases its duration, and long-term medication use can easily cause medicine resistance in diabetic patients. In other words, these diabetic patients have been exposed to adverse conditions for a long time.

BUN and SCr are common biochemical indicators used to evaluate renal function. Previous studies have demonstrated that BUN concentrations have a strong positive correlation with DN incidence risk [24, 25]. Accordingly, a cross-sectional study involving 4,219 T2DM patients found that BUN concentration was an independent risk factor for DN incidence [9]. Data from a community-based study conducted by the Jiangsu Center for Disease Control and Prevention in 2013 indicated that high levels of SCr are strongly related to increased DN incidence risk among T2DM individuals [23]. Similarly, another retrospective study has reported that BUN and SCr levels are positively correlated with DN risk in T2DM patients [10]. In this study, the results of the logistic regression analysis also support the finding that levels of BUN and SCr are strongly associated with a higher risk of DN in patients with T2DM.

NLR and RDW have been recently regarded as important inflammation-related biomarkers and are used to evaluate the occurrence and development of various diseases such as cancers and autoimmune disorders [26–29]. In a cross-sectional study involving 114 T2DM patients, an independent association between NLR and DN was found [30]. Another cross-sectional survey also confirmed that NLR was positively associated with the prevalence of diabetic kidney disease [31]. Regarding RDW, its relationship with renal function has been observed in patients with T2DM [32]. More specifically, RDW was reported to be significantly correlated with increased DN risk and adverse DN prognosis in T2DM patients [33, 34]. Consistent with these aforementioned reports, our study found that NLR and RDW were independent risk factors for DN.

Nomograms are simple and practical tools that are widely used to predict the prognosis and incidence of various diseases. Although several nomogram models have been developed to predict the incidence risk of DN in T2DM subjects [9–11], these nomograms do not consider inflammation-related indicators in their construction. Our study is the first to build a nomogram model that includes inflammation-related indicators, such as NLR and RDW, in predicting the incidence risk of DN among T2DM patients. Hu et al. [10] constructed a nomogram model to predict DN incidence risk, and the C-index of their nomogram was 0.744. Similarly, Jiang et al. [35] established a prognostic nomogram to estimate renal prognosis in DN patients, and the C-index of the model was 0.79. Additionally, Shi et al. [9] reported that the C-index of their DN risk nomogram was 0.807. Unlike our study, the above studies that constructed nomogram models only incorporated biochemical indexes and physical examination results. However, unlike the above studies, besides biochemical indexes and physical examination results, our nomogram model incorporated inflammation-related indicators. Of note, the C-index of our nomogram reached 0.819, and the C-index from internal validation was 0.796. In addition, Jiang et al. [11] recently reported the novel nomogram with a C-index of 0.934 for predicting DN patients. However, they only analyzed a relatively small sample and did not consider inflammation-related indicators. In our study, a relatively moderate sample size and the C-index indicated that our nomogram model displayed moderate and reliable predictive power. Nevertheless, our nomogram has some limitations. First, our nomogram model was constructed using partial indicators; all potential factors influencing DN incidence should be included. Second, this study was a single-center study, and our nomogram needs to be evaluated in T2DM subjects from multiple centers. Finally, although our nomogram achieved good predictive power with internal verification, external validation in T2DM patients from different regions is required.

## 5. Conclusions

In conclusion, we have constructed a novel and simple nomogram with a moderate ability in predicting DN risk in T2DM patients. Based on the evaluation of individual risk, clinicians can provide early medical interventions for T2DM patients. However, our nomogram is not externally validated, and further work is needed to confirm whether individual interventions based on it will decrease the risk of DN incidence and whether it increases clinical usefulness.

## Figures and Tables

**Table 1:** Demographic and clinical characteristics between the DN and NDN groups.

Demographic characteristics	n (%)		
	Total (*n* = 1095)	DN (*n* = 203)	NDN (*n* = 892)
Gender			
Female	472 (43.1)	77 (37.9)	395 (44.3)
Male	623 (56.9)	126 (62.1)	497 (55.7)
Age			
<50	229 (20.9)	33 (16.3)	196 (22.0)
50–69	699 (63.8)	136 (67.0)	563 (63.1)
>69	167 (15.3)	34 (16.7)	133 (14.9)
Smoking			
No	788 (72.0)	131 (64.5)	657 (73.7)
Yes	307 (28.0)	72 (35.5)	235 (26.3)
Consuming alcohol			
No	805 (73.5)	136 (67.0)	669 (75.0)
Yes	290 (26.5)	67 (33.0)	223 (25.0)
Hypertension			
No	638 (58.3)	82 (40.4)	556 (62.3)
Yes	457 (41.7)	121 (59.6)	336 (37.7)
Medicine			
No	233 (21.3)	13 (6.4)	220 (24.7)
Yes	862 (78.7)	190 (93.6)	672 (75.3)
Diabetes duration (years)			
<10	690 (63.0)	83 (40.9)	607 (68.1)
10–20	371 (33.9)	107 (52.7)	264 (29.6)
>20	34 (3.1)	13 (6.4)	21 (2.3)
BMI (kg/m2)			
<24	511 (46.7)	82 (40.4)	429 (48.1)
24–27.99	418 (38.2)	85 (41.9)	333 (37.3)
≥28	166 (15.1)	36 (17.7)	130 (14.6)
FBG (mmol/L)			
<6.1	130 (11.9)	21 (10.3)	109 (12.2)
6.1–6.9	91 (8.3)	13 (6.4)	78 (8.7)
>6.9	874 (79.8)	169 (83.3)	705 (79.1)
PBG (mmol/L)			
<7.8	57 (5.2)	10 (4.9)	47 (5.3)
7.8–11.0	217 (19.8)	44 (21.7)	173 (19.4)
>11.0	821 (75.0)	149 (73.4)	672 (75.3)
HbA1c (%)			
<6.0	59 (5.4)	7 (3.5)	52 (5.8)
6.0–6.9	151 (13.8)	21 (10.3)	130 (14.6)
>6.9	885 (80.8)	175 (86.2)	710 (79.6)
TG (mmol/L)			
<1.7	576 (52.6)	106 (52.2)	470 (52.7)
≥1.7	519 (47.4)	97 (47.8)	422 (47.3)
TC (mmol/L)			
<6.0	959 (87.6)	176 (86.7)	783 (87.8)
≥6.0	136 (12.4)	27 (13.3)	109 (12.2)
HDL (mmol/L)			
<1.0	518 (47.3)	110 (54.2)	408 (45.7)
≥1.0	577 (52.7)	93 (45.8)	484 (54.3)
BUN (mmol/L)			
<3.2	94 (8.6)	11 (5.4)	83 (9.3)
3.2–7.5	856 (78.2)	110 (54.2)	746 (83.6)
>7.5	145 (13.2)	82 (40.4)	63 (7.1)
SCr (*μ*mol/L)			
≤115	977 (89.2)	119 (58.6)	858 (96.2)
>115	118 (10.8)	84 (41.4)	34 (3.8)
UA (*μ*mol/L)			
≤420	905 (82.6)	142 (70.0)	763 (85.5)
>420	190 (17.4)	61 (30.0)	129 (14.5)
Hs-CRP			
≤8	904 (82.6)	155 (76.4)	749 (84.0)
>8	191 (17.4)	48 (23.6)	143 (16.0)
WBC (×10^9^)			
≤10	953 (87.0)	163 (80.3)	790 (88.6)
>10	142 (13.0)	40 (19.7)	102 (11.4)
N			
≤7	970 (88.6)	167 (82.3)	803 (90.0)
>7	125 (11.4)	36 (17.7)	89 (10.0)
L			
≤3.7	1066 (97.4)	198 (97.5)	868 (97.3)
>3.7	29 (2.6)	5 (2.5)	24 (2.7)
NLR			
<1.49	281 (25.7)	26 (12.8)	255 (28.6)
1.49–1.99	274 (25.0)	41 (20.2)	233 (26.1)
2.00–2.84	267 (24.4)	50 (24.6)	217 (24.3)
>2.84	273 (24.9)	86 (42.4)	187 (21.0)
RDW (%)			
<12.0	292 (26.7)	35 (17.2)	257 (28.8)
12.0–12.4	275 (25.1)	47 (23.2)	228 (25.6)
12.5–13.0	266 (24.3)	55 (27.1)	211 (23.6)
>13.0	262 (23.9)	66 (32.5)	196 (22.0)

BMI, body mass indices; FBG, fasting blood glucose; PBG, postprandial blood glucose; HbA1c, glycosylated hemoglobin A1c; TG, triglycerides; TC, total cholesterol; HDL, high‐density lipoprotein cholesterol; BUN, blood urea nitrogen; SCr, serum creatinine; UA, uric acid; Hs‐CRP, high sensitivity C‐reactive protein; WBC, white blood cell; N, neutrophil; L, lymphocyte; NLR, neutrophil to lymphocytes ratio; RDW, red blood cell distribution width.

**Table 2:** Screening out predictive factors for DN incidence risk in patients with T2DM by logistic regression.

Intercept and variable	Logistic regression				
	*β*-coefficient	Z-value	*P* value	OR	95% CI
Intercept	−4.766	−6.282	<0.001^*∗∗∗*^	0.009	0.002–0.035
Gender = male	−0.546	−2.199	0.028^*∗*^	0.579	0.353–0.937
Age = 50–69	−0.415	−1.535	0.125	0.660	0.391–1.131
Age > 69	−0.806	−2.259	0.024^*∗*^	0.447	0.220–0.896
Smoking = yes	0.366	1.262	0.207	1.443	0.817–2.555
Consuming alcohol = yes	0.562	1.932	0.053	1.754	0.993–3.107
Hypertension = yes	0.397	1.965	0.049^*∗*^	1.487	1.001–2.211
Medicine = yes	1.152	3.286	0.001^*∗∗*^	3.163	1.641–6.538
Diabetes duration = 10–20	0.855	3.965	<0.001^*∗∗∗*^	2.350	1.545–3.602
Diabetes duration > 20	0.997	2.161	0.031^*∗*^	2.709	1.070–6.587
BMI = 24–27.99	0.382	1.763	0.078	1.466	0.958–2.245
BMI ≥ 28	0.574	1.993	0.046^*∗*^	1.775	1.002–3.108
HbA1c = 6–6.9	−0.283	−0.501	0.616	0.753	0.256–2.395
HbA1c > 6.9	0.624	1.284	0.199	1.866	0.765–5.234
TC ≥ 6	0.262	0.877	0.380	1.299	0.712–2.303
HDL ≥ 1.0	−0.258	−1.296	0.195	0.772	0.522–1.142
BUN = 3.2–7.5	−0.062	−0.168	0.867	0.940	0.473–2.030
BUN > 7.5	0.958	2.095	0.036^*∗*^	2.608	1.084–6.578
SCr > 115	2.328	7.551	<0.001^*∗∗∗*^	10.256	5.655–18.993
UA > 420	−0.461	−1.679	0.093	0.631	0.362–1.066
WBC > 10	0.695	1.636	0.102	2.003	0.851–4.524
*N* > 7	−0.877	−1.787	0.074	0.416	0.159–1.095
NLR = 1.49–1.99	0.562	1.857	0.063	1.754	0.974–3.206
NLR = 2.00–2.84	0.697	2.352	0.019^*∗*^	2.008	1.131–3.630
NLR > 2.84	0.986	3.047	0.002^*∗∗*^	2.682	1.428–5.099
RDW = 12.0–12.4	0.370	1.280	0.200	1.447	0.824–2.564
RDW = 12.5–13.0	0.721	2.499	0.012^*∗*^	2.056	1.175–3.648
RDW > 13.0	0.729	2.582	0.009^*∗∗*^	2.073	1.198–3.633

Note: “∗∗∗”indicates *P* < 0.001, “∗∗”indicates *P* < 0.01, “∗” indicates *P* < 0.05, BMI, body mass indices; HbA1c, glycosylated hemoglobin A1c; TC, total cholesterol; HDL, high‐density lipoprotein cholesterol; BUN, blood urea nitrogen; SCr, serum creatinine; UA, uric acid; WBC, white blood cell; N, neutrophil; NLR, neutrophil to lymphocytes ratio; RDW, red blood cell distribution width.

## Data Availability

The data used to support the findings of this study are available from the corresponding author upon request.
